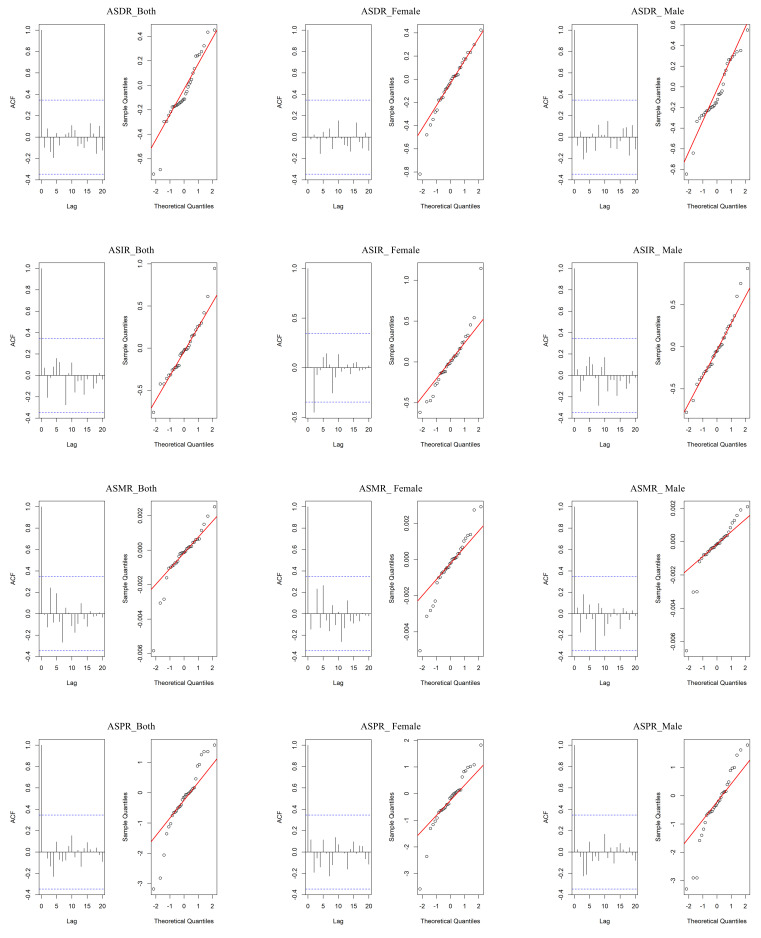# Analysis and comparison of the trends in burden of congenital musculoskeletal and limb anomalies in China and worldwide from 1990 to 2021

**DOI:** 10.1097/MD.0000000000049244

**Published:** 2026-06-12

**Authors:** Min-cheng Zou, Wen-dong Liu, Xiao-dong Wang, Guang-hao Su, Ya Liu

**Affiliations:** a Department of Orthopedics, Children’s Hospital of Soochow University, Suzhou, Jiangsu Province, China; b Pediatric Clinical Research Institute, Children’s Hospital of Soochow University, Suzhou, China.

**Keywords:** burden of disease, congenital musculoskeletal and limb anomalies, DALYs, disease predictions, incidence, mortality, prevalence

## Abstract

Congenital musculoskeletal and limb anomalies (CMLA) are common birth defect worldwide. This study aims to describe temporal trends of age- and gender-specific burden of CMLA in China (1990–2021), including incidence, prevalence, mortality, and disability-adjusted life years (DALYs), and to compare these with the global burden, and to predict burden changes in the next 15 years in China. Using the open access data of the Global Burden of Disease database (1990–2021), the incidence, prevalence, mortality, and DALYs of CMLA in China and worldwide were extracted. Age-standardized rates were calculated. Joinpoint regression analysis was used to calculate the average annual percentage change (AAPC) to evaluate time trends. Auto-regressive integrated moving average model was used to predict the burden in China in 2022 to 2036. Decomposition analysis was applied to evaluate the contribution of aging, population, and epidemiological changes to the change in disease burden. From 1990 to 2021, absolute numbers of incidence, prevalence, mortality, and DALYs of CMLA in China decreased by 51.54%, 16.79%, 83.02%, and 29.04%, respectively, far exceeding the global level (−3.32%, +19.13%, −28.05%, and −1.17%). The age-standardized mortality rate (ASMR) and age-standardized DALY rate (ASDR) in China decreased significantly (AAPC = −4.531% and −1.055%, respectively), and the age-standardized prevalence rate (ASPR) also decreased (AAPC = −0.584%). The age-standardized incidence rate (ASIR) was relatively stable (AAPC = 0.047%). The global ASPR, ASMR, and ASDR showed a downward trend, while ASIR was basically stable. China’s burden shows a significant age shift (ages ≤29 decreases, ≥30 increases) and gender differences (male burden exceeded female across indicators). Auto-regressive integrated moving average forecasts that ASPR, ASDR, and ASMR will continue declining over the next 15 years, while female ASIR may increase slightly. Decomposition analysis showed that epidemiological changes were the main factors driving the age-standardized rate decline, and population growth hindered the decline. China has made remarkable achievements in reducing the disease burden of CMLA, at a time when the decline in the global burden has been slow and uneven. The burden in China showed the characteristics of migration from children to adults, with persistent gender differences.

## 1. Background

Congenital musculoskeletal and limb anomalies (CMLA) are types of birth defects that are common in the world, which seriously affect the health and quality of life of children and even their adulthood. The World Health Organization estimates that congenital anomalies are among the leading causes of neonatal death and long-term disability in children.^[1]^

CMLA has a significant impact on patients and their families. These deformities may lead to severe physical dysfunction, affecting physical activity ability, daily life, and psychological well-being. On the contrary, the required long-term medical needs and social support can impose a heavy financial burden on families and society. Therefore, it is essential to understand the global epidemiological characteristics and time trends of such diseases and develop targeted prevention strategies. To reduce mortality from CMLA, countries affected by CMLA need to systematically implement evidence-based prevention and control strategies and rigorously evaluate potential strategies and their cost-effectiveness to help further reduce mortality. Therefore, tracking temporal trends in the burden of CMLA has become essential for health strategies.

The global burden of CMLA is significantly heterogeneous, with different geographic regions, ethnic groups, and socioeconomic strata strongly influencing its prevalence.^[2]^ Current reports on the burden of CMLA in Global Burden of Disease (GBD) studies mainly focus on macroscopic assessments at global and regional levels. Existing studies estimated the global trend and attributable risk of CMLA from 1990 to 2021, analyzed its relationship with socioeconomic development, and predicted the future burden of CMLA.^[3]^ However, most of these studies are conducted from a global perspective, lack in-depth research on the heterogeneity issues between different countries and regions, and ignore the specific situation of specific countries.

The absolute number of CMLA cases is concentrated in large population countries, and China is one of the countries with the highest burden due to its large population base.^[3]^ Therefore, as the most populous country in the world, the disease burden of CMLA in China remains to be fully elucidated. Although there have been some relevant studies on the burden of congenital malformations in China,^[4,5]^ these analyses have not yet specifically and comprehensively investigated the progress of CMLA in the Chinese population. Therefore, this study examines the epidemiology and disease burden of CMLA in China and globally based on the latest GBD 2021 data. This study aims to provide a detailed overview of the incidence, prevalence, mortality, and disability-adjusted life years (DALYs) of CMLA in China to measure the disease burden. Joinpoint regression analysis was used to explore the temporal trends of CMLA and to in-depth examine the changes in the burden of CMLA over the past 30 years from the perspective of age and gender. The incidence, prevalence, mortality, and DALYs of CMLA in China in the next 15 years were also projected. These insights are expected to help deepen the understanding of the overall burden of CMLA in China and facilitate the development of targeted prevention strategies and equitable allocation of public health resources.

## 2. Methods

### 2.1. Data source

The data used in this study were extracted from the GBD 2021 dataset, a comprehensive database of incidence, prevalence, and mortality of more than 300 diseases and injuries, disaggregated by age and sex across 204 countries and territories. The GBD dataset was drawn from a variety of sources and included 46,749 cohort studies, randomized controlled trials, civilian surveys, and other studies.^[6]^

The incidence, prevalence, mortality, and DALYs of CMLA in China and the world from 1990 to 2021 were screened from the GBD database through the Global Health Data Exchange (GHDx) query tool (http://ghdx.healthdata.org/gbd-results-tool) as indicators to measure the burden of disease.

In the GBD database, CML abnormalities are classified as “Non-communicable diseases,” specifically “Other non-communicable diseases.” It is located under the “Congenital birth defects” category of the level 4 directory. CML abnormalities are defined as abnormalities of the muscle or skeletal system that are present at birth and not due to a defined chromosomal syndrome. These abnormalities were further divided into 3 subcategories: polydactyly and syndactyly, limb reduction defects, and all other CML abnormalities. Polydactyly and syndactyly correspond to International Classification of Diseases, 10th Revision codes Q69 to Q70, limb-reduction defects to Q71 to Q73, and other CML abnormalities to Q65 to Q68 and Q74 to Q79.^[3,7]^

All summary data are publicly accessible through GHDx, thus ensuring transparency and reproducibility of the methodology. Furthermore, given that the data from the 2021 GHDx platform have been made public, this study does not require approval from the ethics committee and thus has been granted an exemption. Guidelines for accurate and transparent reporting of health assessments were followed in this study.

### 2.2. Statistical analysis

Age-standardized incidence rate (ASIR), age-standardized prevalence rate (ASPR), age-standardized mortality rate (ASMR), and age-standardized DALY rate (ASDR), as well as the crude incidence rate (CIR), crude prevalence rate (CPR), crude mortality rate, and crude DALY rate (CDR), were presented in this study. Age standardization is essential when comparing populations with different age structures or examining changes in age distribution over time within the same population. We used standardized global populations from the GBD database to determine age-standardized rates (ASRs).

The average annual percentage change (AAPC) with the corresponding 95% confidence interval (95% CI) was calculated using Joinpoint software (National Cancer Institute, Rockville) to determine trends in the burden of the disease.^[8]^ A regression model can be fitted with log age-standardized measures, that is, ln(y) = α + βx + ε, where y represents the corresponding age-standardized measure and x represents the calendar year. The AAPC was calculated using the formula 100 × (exp(β) − 1), and the 95% CI can also be calculated from the model. If the 95% CI of the corresponding AAPC estimate was >0, the age-standardized indicator showed an upward trend. If <0, it showed a downward trend. If 0 is included, it shows a stable trend.

Taking into account demographic denominator differences between genders, we calculated age- and sex-stratified corrected incidence, prevalence, mortality, and DALY rates for the 1990 and 2021 CMLA in China. We extracted the number of incidence, prevalence, deaths, DALYs, and the corresponding population estimates. Rate (corrected) = number of cases/population. These ratios were then used to calculate male-to-female ratios for each age group. This eliminates the effect of differences in population size and enables direct comparison of disease burden between sexes after age stratification.

To characterize the shift in CMLA burden across age groups between 1990 and 2021, we calculated the percentage change in age‑specific prevalence rates for each age group. The percentage change = ([Rate_2021 − Rate_1990]/Rate_1990) × 100%. A positive value indicates an increase in burden over the 3 decades, whereas a negative value indicates a decrease. The median percent change in prevalence and 95% uncertainty intervals (UI) were calculated from 100,000 Monte Carlo simulations.

The R statistical software program (version 4.4.2) and Joinpoint software program (version 5.4.0.0) were used for statistical analysis and visualization of the data. A *P* value <.05 was considered statistically significant. The significance level for all AAPCs and their 95% CI was set at α = 0.05, meaning that the trend was considered statistically significant (*P* < .05) if the 95% CI did not include zero.

### 2.3. Auto-regressive integrated moving average model

The auto-regressive integrated moving average (ARIMA) model is used to predict the disease burden trend of CMLA in the next 15 years. ARIMA is a forecasting method for time series data that combines autoregressive and moving average components with difference (d) to stabilize the series. The ARIMA model has 3 key parameters, namely, p, d, and q, where p represents the order of the autoregressive term, d represents the degree of difference, and q represents the order of the moving average term. At the heart of ARIMA is the difference process, which transforms non-stationary sequences into stationary sequences, thus allowing more efficient modeling.

While the Augmented Dickey-Fuller (ADF) test is also widely used to test stationarity, the Kwiatkowski–Phillips–Schmidt–Shin (KPSS) test uses stationarity as the null hypothesis, making it more conservative in choosing the difference order. The KPSS test has been shown to yield higher prediction accuracy than ADF-based differential selection when using automated ARIMA modeling.^[9]^ In addition, given the relatively short time span of the GBD data (1990–2021), the KPSS test is more reliable in detecting nonstationarity under small sample conditions.^[10]^ Therefore, the KPSS test was used to assess the stationarity of the original time series. The KPSS test used the null hypothesis of stationarity (H_0_: the sequence was stationary), and a *P* value >.05 indicated that the sequence was stationary. If the original sequence was found to be nonstationary (*P* < .05), the first difference was used, and the KPSS test was repeated until stationary (*P* > .05) was achieved.

The best ARIMA model was selected by fitting using the “auto.arima()” function in the “forecast” package in R (version 4.4.2). The order of the difference d was determined using the KPSS test, followed by a stepwise search of p and q to minimize the corrected Akaike information criterion. To verify the adequacy of the final model, we performed the Ljung-Box test on the model residuals (lag = 10) to check for white noise (*P* > .05). Residual diagnostics were further performed by examining ACF plots and quantile (Q-Q) plots to confirm mean zero, no residual autocorrelation, and approximate normality.

### 2.4. Decomposition analysis

The decomposition analysis was used to more accurately and intuitively show the impact of aging, population, and epidemiological factors on the changes in incidence, prevalence, mortality, and DALY of CMLA from 1990 to 2021, in order to understand how these factors affect the disease burden of CMLA individually and jointly. In this paper, R (version 4.4.2) was used for decomposition analysis, and the “ggplot2” package was used to visualize the prediction results.

To account for the uncertainty inherent in GBD estimation, we propagated the 95% UI of the input parameters into the decomposition analysis using Monte Carlo simulation methods. For each age-sex group, a lognormal distribution (standard error = [UI_upper − UI_lower]/[2 × 1.96]) was assumed. Das Gupta decomposition was repeated with 1000 randomly selected samples, with the median as the effect estimate and the 2.5th and 97.5th percentiles as the 95% UI.

## 3. Results

### 3.1. Description of the burden of CMLA in China and globally

#### 3.1.1. Incidence of CMLA in China and worldwide

The incidence of CMLA in China decreased from 492,326 cases (95% CI: 337,691–701,398) in 1990 to 238,561 cases (95% CI: 169,545–338,147) in 2021, with a cumulative decrease of 51.54%. However, the global incidence of CMLA decreased from 2,521,672 cases (95% CI: 1,793,996–3,516,239) in 1990 to 2,437,890 cases (95% CI: 1,737,730–3,355,568) in 2021, with a reduction of only 3.32%. The global ASIR increased from 39.352 (95% CI: 27.996–54.873) in 1990 to 39.408 (95% CI: 28.09–54.242) in 2021. In China, ASIR decreased from 44.532 (95% CI: 30.545–63.443) in 1990 to 45.01 (95% CI: 31.989–63.799) in 2021. At the same time, the AAPC of incidence in China increased by 0.047% (95% CI: −0.120 to 0.213) from 1990 to 2021, while the AAPC of incidence increased by 0.004% (95% CI: −0.020 to 0.027) globally (Table 1).

**Table 1:** Disease burden of CMLA in China and worldwide in1990 and 2021.

Location	Measure	1990		2021		1990–2021 AAPC
		All-age cases	Age-standardized rates per 100,000 people	All-age cases	Age-standardized rates per 100,000 people	
		n (95% CI)	n (95% CI)	n (95% CI)	n (95% CI)	n (95% CI)
China	Incidence	492,326 (337,691–701,398)	44.532 (30.545–63.443)	238,561 (169,545–338,147)	45.01 (31.989–63.799)	0.047 (−0.120 to 0.213)
	Prevalence	3,740,926 (3,014,594–4,627,168)	316.363 (254.462–391.762)	3,112,684 (2,565,594–3,820,090)	266.13 (219.542–325.376)	−0.584 (−0.669 to −0.499)
	Deaths	1402 (755–2324)	0.124 (0.066–0.206)	238 (152–319)	0.03 (0.018–0.042)	−4.531 (−4.730 to −4.332)
	DALYs	649,473 (449,791–888,410)	55.29 (38.237–75.47)	460,892 (307,116–655,005)	40.194 (27.245–57.953)	−1.055 (−1.172 to −0.938)
Global	Incidence	2,521,672 (1,793,996–3,516,239)	39.352 (27.996–54.873)	2,437,890 (1,737,730–3,355,568)	39.408 (28.09–54.242)	0.004 (−0.020 to 0.027)
	Prevalence	15,570,721 (12,578,432–19,071,446)	273.498 (221.492–333.308)	18,549,408 (15,159,833–22,636,857)	249.06 (203.507–304.124)	−0.313 (−0.343 to −0.283)
	Deaths	18,902 (10,324–30,381)	0.304 (0.167–0.486)	13,600 (10,506–18,111)	0.209 (0.16–0.28)	−1.200 (−1.266 to −1.132)
	DALYs	3,903,854 (2,777,029–5,312,390)	65.87 (46.754–89.222)	3,858,032 (2,860,408–5,050,546)	54.32 (40.695–71.093)	−0.618 (−0.662 to −0.575)

All-age cases and age-standardized incidence, prevalence, mortality, and DALY rates and corresponding AAPC of CMLA in China and globally in 1990 and 2021.

AAPC = average annual percentage change, CI = confidence interval, CMLA = congenital musculoskeletal and limb anomalies, DALYs = disability-adjusted life years.

#### 3.1.2. Prevalence of CMLA in China and worldwide

In terms of prevalence, the number of CMLA cases in China decreased from 3,740,926 (95% CI: 3,014,594–4,627,168) in 1990 to 3112,684 (95% CI: 2,565,594–3,820,090) in 2021, with a cumulative decline of 16.79%. However, the prevalence of CMLA increased from 15,570,721 (95% CI: 12,578,432–19,071,446) in 1990 to 18,549,408 (95% CI: 15,159,833–22,636,857) in 2021, with a cumulative increase of 19.13%. The global ASPR increased from 273.498 cases per 100,000 population (95% CI: 221.492–333.308) in 1990 to 249.06 cases per 100,000 population (95%CI: 203.507–304.124) in 2021. ASPR in China decreased from 316.363 cases (95% CI: 254.462–391.762) in 1990 to 266.13 cases (95%CI: 219.542–325.376) in 2021. Meanwhile, the global AAPC of prevalence decreased by 0.313% (95% CI: −0.343 to −0.283) from 1990 to 2021, and in China, this number decreased by 0.584% (95% CI: −0.669 to −0.499; Table 1).

#### 3.1.3. Mortality of CMLA in China and worldwide

In 2021, CMLA caused 13,600 deaths (95% CI: 10,506–18,111) globally, a decrease of 28.05% compared with 1990. In China, the mortality rate decreased by 83.02% from 1990 to 2021. The global ASMR decreased from 0.304 (95% CI: 0.167–0.486) per 100,000 population in 1990 to 0.209 (95% CI: 0.16–0.28) per 100,000 population in 2021. In China, ASMR decreased from 0.124 per 100,000 population (95% CI: 0.066–0.206) in 1990 to 0.03 per 100,000 population (95% CI: 0.018–0.042) in 2021. The global AAPC of mortality decreased by 1.200% (95% CI: −1.266 to −1.132) from 1990 to 2021, while in China, it decreased by 4.531% (95% CI: −4.730 to −4.332; Table 1).

#### 3.1.4. DALYs of CMLA in China and worldwide

Globally, the DALYs for CMLA were 3,903,854 (95% CI: 2,777,029–5,312,390) in 1990 and 3,858,032 (95% CI: 2,860,408–5,050,546) in 2021, representing a 1.17% decrease compared with 1990. In China, the DALYs decreased by 29.04% from 1990 to 2021. The ASDR globally decreased from 65.87 (95% CI: 46.754–89.222) per 100,000 population in 1990 to 54.32 (95% CI: 40.695–71.093) per 100,000 population in 2021. In China, the ASDR decreased from 55.29 (95% CI: 38.237–75.47) per 100,000 population in 1990 to 40.194 (95% CI: 27.245–57.953) per 100,000 population in 2021. Meanwhile, the AAPC of the DALYs globally decreased by 0.618% (95% CI: −0.662 to −0.575) from 1990 to 2021, while it decreased by 1.055% (95% CI: −1.172 to −0.938) in China (Table 1).

### 3.2. Joinpoint regression analysis of the burden of CMLA in China and worldwide

**Figure 1.**
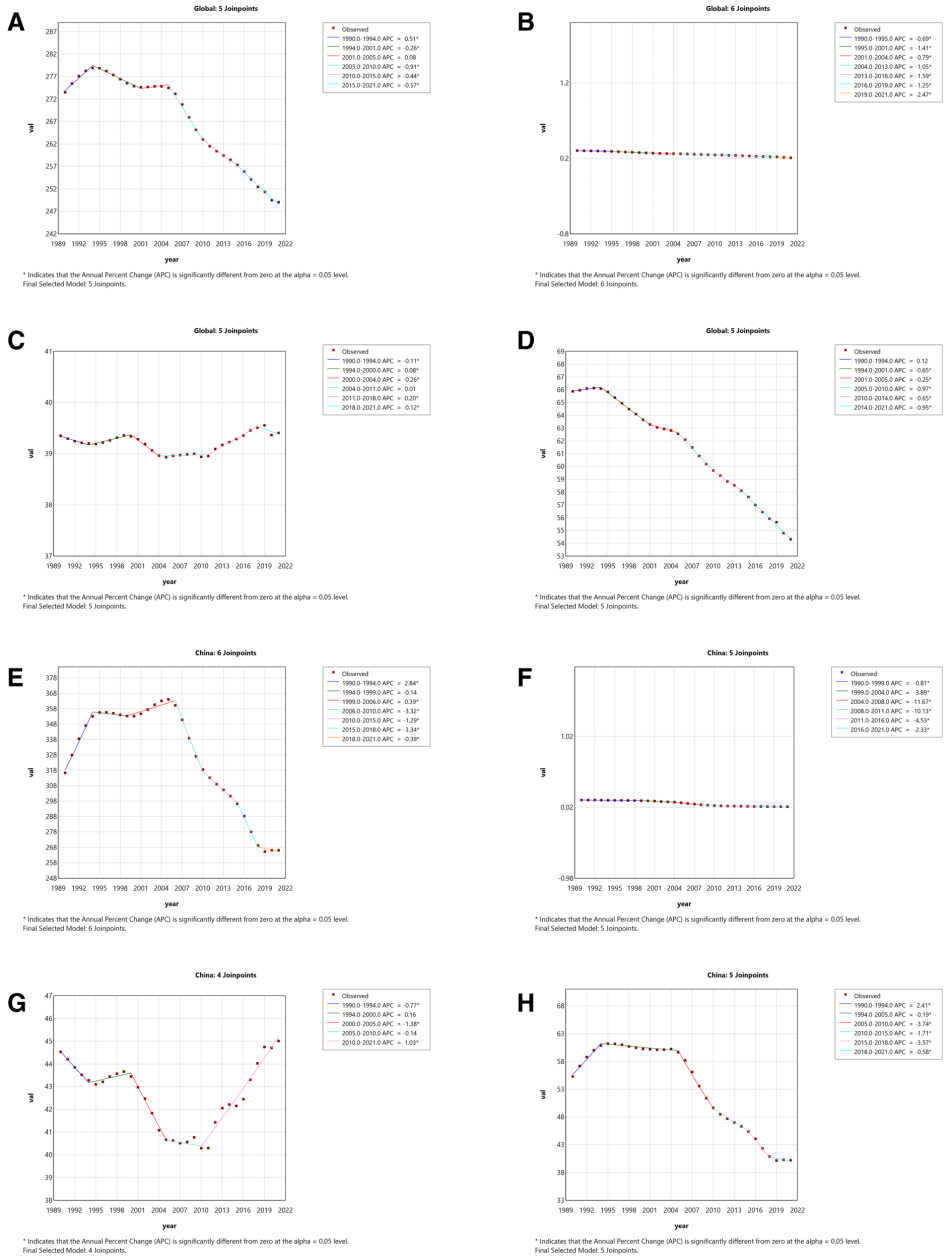
The APC of ASIR, ASPR, ASMR, and ASDR of CMLA in China and globally from 1990 to 2021 (*means *P* values < .05 and significant results). (A) Global-ASPR, (B) global-ASMR, (C) global-ASIR, (D) global-ASDR, (E) China-ASPR, (F) China-ASMR, (G) China-ASIR, (H) China-ASDR. ASDR = age-standardized DALY rate, ASPR = age-standardized prevalence rate, ASIR = age-standardized incidence rate, ASMR = age-standardized mortality rate, CMLA = congenital musculoskeletal and limb anomalies, DALYs = disability-adjusted life years.

The Joinpoint regression analysis of ASIR, ASPR, ASMR, and DALYs for CMLA in China and worldwide from 1990 to 2021 is depicted in Figure 1. The annual percentage change (APC) of CMLA ASDR and ASPR in China showed a significant upward trend from 1990 to 1995. In the following decade (1996–2005), China’s ASDR declined slowly, while its ASPR increased. Both of them decreased significantly from 2006 to 2019, and the downward trend slowed down significantly after 2019 (Fig. 1E, 1H). Globally, the ASDR and ASPR of CMLA showed a significant downward trend after 1995 (*P* < .05), after an increase from 1990 to 1995 (Fig. 1A, 1D).

The ASIR of CMLA in China and the world showed a downward trend from 1990 to 1994, and after a slow increase from 1995 to 1999, it showed a significant decline from 2000 to 2005. It was flat from 2006 to 2010. From 2010 to 2019, both China’s ASIR and the global ASIR showed a significant increase, and China’s ASIR curve was steeper. From 2019 to 2021, while China’s ASIR continued to rise, the global ASIR experienced a decline (Fig. 1C, 1G).

From 1990 to 2021, the ASMR of CMLA in China and globally showed a slow downward trend (*P* < .05; Fig. 1B, 1F).

### 3.3. Trends in the burden of CMLA in China and globally

**Figure 2.**
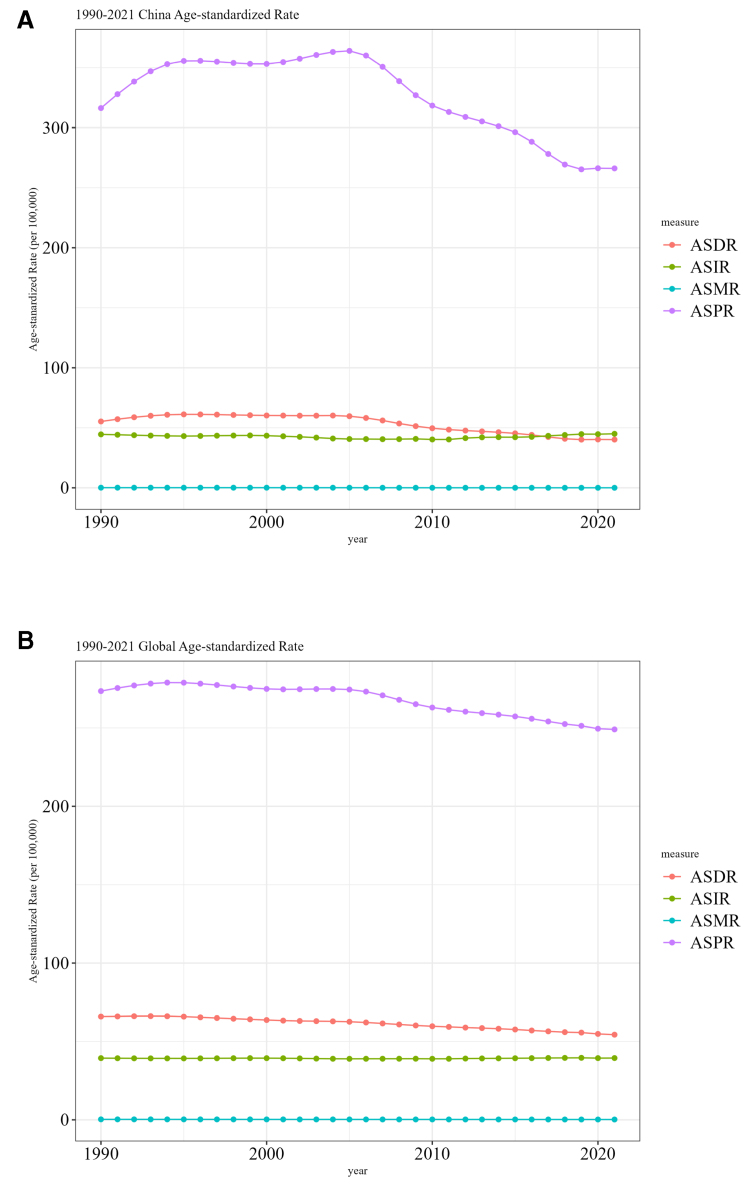
Trend comparison of ASIR, ASPR, ASMR, and ASDR of CMLA in China and globally from 1990 to 2021. (A) China_burden_trend; (B) Global_burden_trend. ASDR = age-standardized DALY rate, ASPR = age-standardized prevalence rate, ASIR = age-standardized incidence rate, ASMR = age-standardized mortality rate, CMLA = congenital musculoskeletal and limb anomalies, DALYs = disability-adjusted life years.

From 1990 to 2021, the ASDR of CMLA in China and globally showed a slight downward trend, and the decline in China was greater. Meanwhile, the ASPR trend of China’s CMLA showed a significant upward trend from 1990 to 2005, and a significant downward trend after 2005. In contrast, the ASPR of global CMLA showed a flat trend from 1990 to 2005, and then showed a gradual downward trend after 2005. In addition, both ASIR and ASMR of CMLA in China were basically flat from 1990 to 2021, while the ASIR of CMLA worldwide showed a slight upward trend, while the ASMR was basically flat (Fig. 2).

### 3.4. Burden of CMLA in different age groups in 1990 and 2021 in China and worldwide

**Figure 3.**
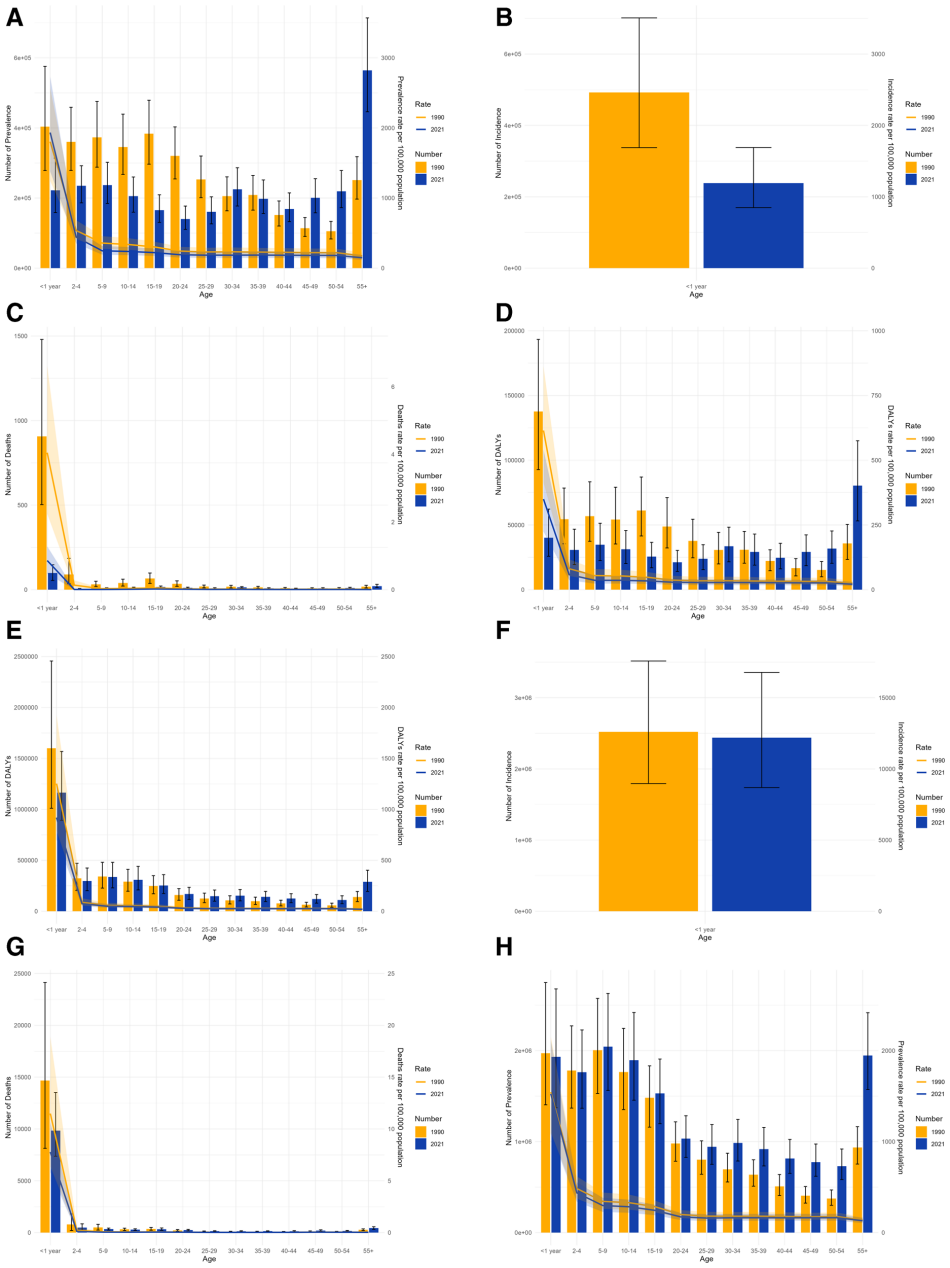
Comparison of the incidence, prevalence, deaths, and DALYs counts, along with their crude rates, by age group in China and globally from 1990 to 2021. (A) Prevalent cases and CPR (China); (B) incident cases and CIR (China); (C) death cases and CMR (China); (D) DALYs counts and CDR (China); (E) prevalent cases and CPR (global); (F) incident cases and CIR (global); (G) Death cases and CMR (global); (H) DALYs counts and CDR (global). Bar charts represent counts (with 95% CI); lines represent crude rates. CI = confidence interval, CDR = crude DALY rate, CIR = crude incidence rate, CPR = crude prevalence rate, CMR = crude mortality rate, DALYs = disability-adjusted life years.

Figure 3 compares the incidence, prevalence, mortality, DALYs, and their crude rates of CMLA in different age groups in China and globally in 1990 and 2021. According to the incidence results, the incidence number of CMLA in children below 1 year old in China in 2021 decreased significantly compared with that in 1990, while the decline was weak at the global level (Fig. 3B, 3F). From the perspective of prevalence, the crude prevalence of CMLA in China and the world in 1990 and 2021 both showed a downward trend with age, and the decline was particularly significant in the age group of ≤ 4 years old (Fig. 3A). Similar trends were observed for crude mortality rate and CDR both in China and globally. The number of deaths was concentrated in children below 1 year old, and the number of deaths in 2021 was significantly lower than that in 1990 (Fig. 3C, 3G). Interestingly, in 2021, the number of prevalence and DALYs in the age group under 29 years old in China decreased significantly compared with 1990, while the number of prevalence and DALYs in the age group over 30 years old increased, and the increase was more pronounced with age (Fig. 3A, 3D). At the global level, compared with 1990, the number of cases in all age groups and DALYs over 10 years old showed an increasing trend in 2021, and the increase in the number of cases was more significant with the increase of age (Fig. 3E, 3H).

From 1990 to 2021, the decline rate of the prevalence in all age groups in China increased first and then decreased. The largest decrease (−31.0%) was observed in the 5 to 9 years old group, and the change was statistically significant. Then, the decline gradually decreased to −15.0% in the group aged over 55 years. Similarly, the global pattern showed a larger decline in childhood and a slower decline in adulthood. Monte Carlo simulations showed that the change did not reach statistical significance (95% UI included zero; Table 2).

**Table 2:** Percentage change in age‑specific prevalence of CMLA between 1990 and 2021 in China and globally.

Age group (yr)	China prevalence, % change (95% UI)	Global prevalence, % change (95% UI)
<1	+7.0 (−36.9, 85.0)	−1.4 (−40.2, 63.9)
2–4	−17.2 (−40.9, 17.5)	−9.6 (−36.8, 29.9)
5–9	−31.0 (−51.9, −0.7)	−13.5 (−40.7, 26.6)
10–14	−29.5 (−50.6, 1.3)	−13.7 (−40.4, 25.0)
15–19	−26.7 (−48.1, 3.3)	−14.1 (−38.4, 19.6)
20–24	−21.0 (−43.9, 11.1)	−13.0 (−36.8, 20.0)
25–29	−19.4 (−42.8, 13.6)	−11.5 (−36.5, 23.3)
30–34	−20.3 (−43.7, 12.5)	−9.5 (−35.1, 26.0)
35–39	−18.4 (−42.3, 15.3)	−9.4 (−34.9, 26.1)
40–44	−18.4 (−42.2, 15.2)	−8.2 (−33.8, 27.4)
45–49	−17.8 (−42.0, 16.4)	−6.2 (−32.6, 30.3)
50–54	−18.0 (−42.2, 16.0)	−7.2 (−33.4, 28.9)
55+	−15.0 (−39.9, 20.3)	−6.0 (−31.5, 28.7)

Negative values indicate a decrease in prevalence, and positive values indicate an increase.

CMLA = congenital musculoskeletal and limb anomalies, UI = uncertainty interval.

### 3.5. Gender differences in the burden of CMLA in different age groups in China and globally

**Figure 4.**
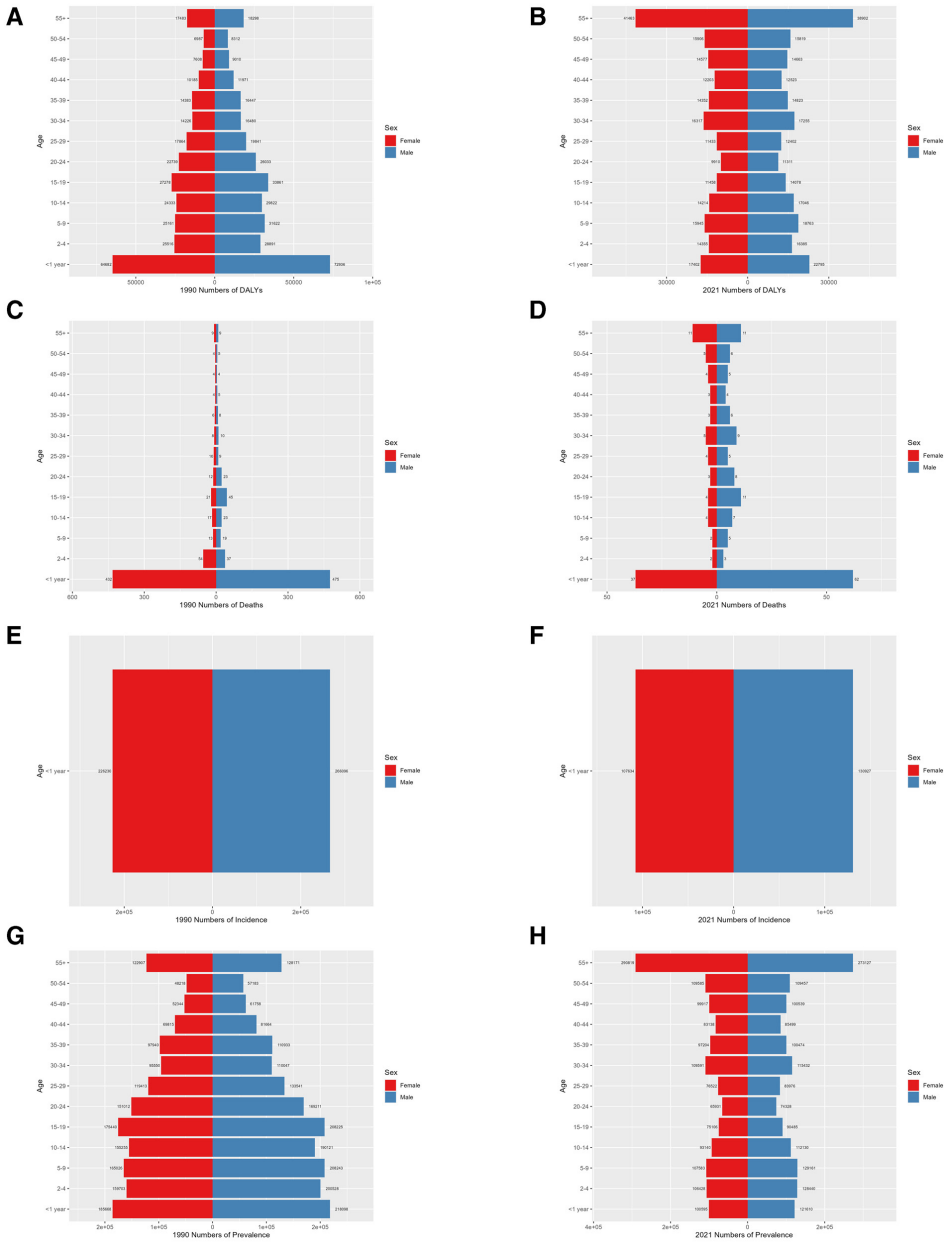
Comparison of the number of incidence, prevalence, mortality, and DALYs of CMLA in males and females of different age groups in China in 1990 and 2021. (A, B) DALYs; (C, D) mortality; (E, F) incidence; (G, H) prevalence. CMLA = congenital musculoskeletal and limb anomalies, DALYs = disability-adjusted life years.

Figure 4 shows the incidence, prevalence, mortality, and DALYs of CMLA in males and females of different age groups in China in 1990 and 2021. The number of incidence, prevalence, deaths, and DALYs of CMLA in males were higher than those in females in all age groups. In 1990, the peak of DALY appeared in the age group of <1 year in both males and females, and DALYs showed a downward trend with age. The peak of incidence in the non-children population appeared in the age group of 15 to 19 years. In 2021, the peak of DALY appeared in the 55+ years old group for both sexes in China, and the DALY of other age groups was basically the same, although there were differences (Fig. 4A and 4B). In 1990 and 2021, the number of deaths in both males and females in China was concentrated in the age group <1 year old, and the proportion of deaths <1 year old decreased in 2021 due to the significant reduction in mortality (Fig. 4C and 4D). In 1990, the number of Chinese males and females with CMLA was basically the same in all age groups of 19 years old and below, and the number of Chinese males and females with CMLA in all age groups above 19 years old decreased with age (Fig. 4G). Except for the 15 to 29 age group, which had a lower number of cases, the distribution of cases in the other age groups was similar in both men and women in China in 2021 (Fig. 4H). After standardization according to age stratification, indicators that differed among age groups still existed (Table S1, Supplemental Digital Content 1).

**Figure 5.**
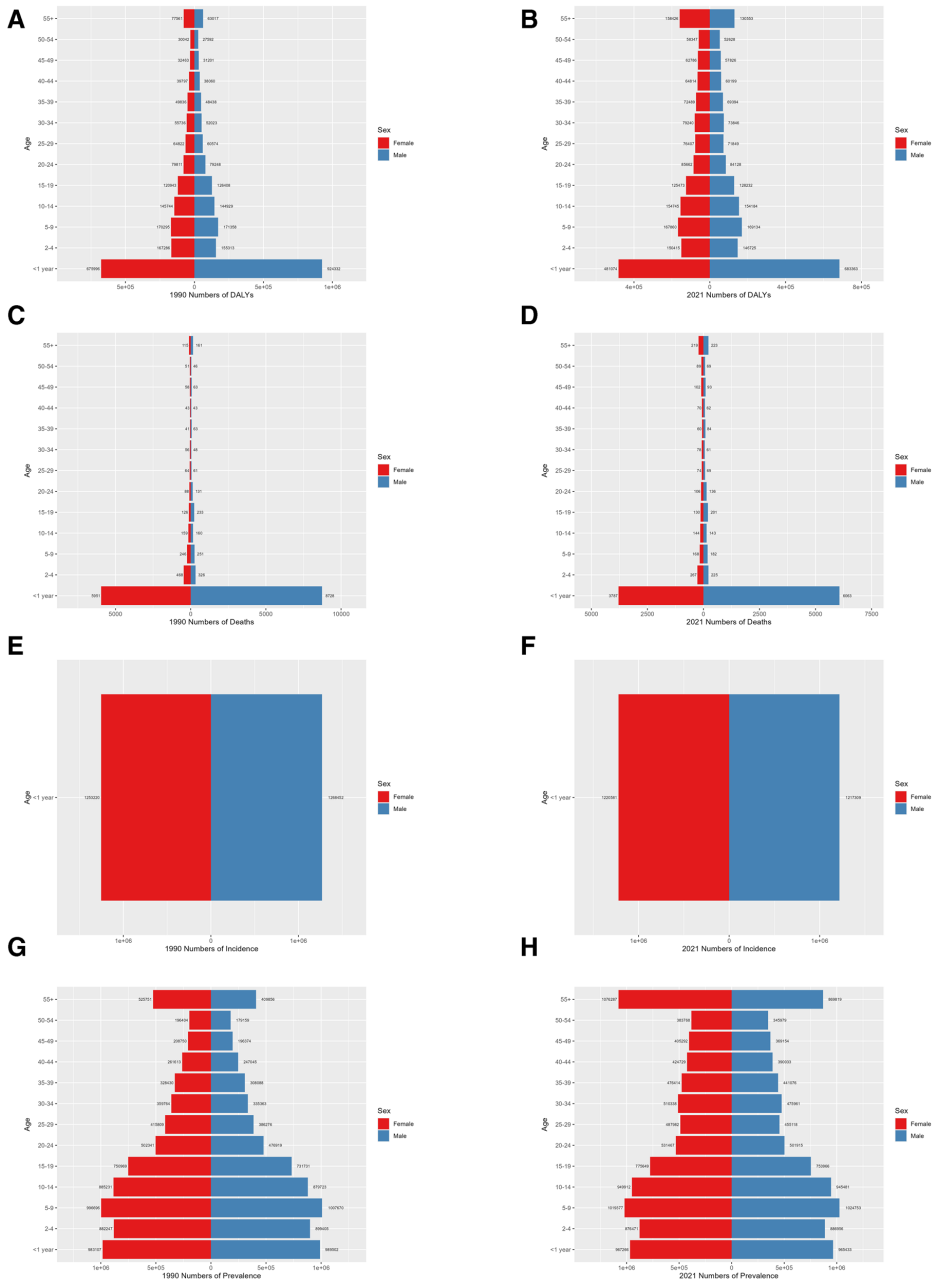
Comparison of the number of incidence, prevalence, mortality, and DALYs of CMLA in males and females of different age groups globally in 2019 and 2021. (A, B) DALYs; (C, D) mortality; (E, F) incidence; (G, H) prevalence. CMLA = congenital musculoskeletal and limb anomalies, DALYs = disability-adjusted life years.

Figure 5 shows the incidence, prevalence, mortality, and DALYs of CMLA for males and females in different age groups worldwide in 1990 and 2021. Both incidence and prevalence numbers of CMLA in males were higher than those in females in all age groups, which was consistent with the situation in China, while the number of deaths and DALYs was similar between males and females. In 1990 and 2021, DALYs and deaths peaked in the age group below 1 year for both males and females and showed a downward trend with the increase in age (Fig. 5A–5D). In 1990 and 2021, the prevalence numbers of CMLA in males and females were similar in all age groups of 10 years old and younger, while the prevalence numbers of CMLA in females were higher than those in males in all age groups of 10 years old and older. In 1990, the number of cases of CMLA peaked in the age group of <1 year old and 5 to 9 years old, and the number of cases decreased with age. In 2021, the number of cases of CMLA peaked in the age group of <1 year old, 5 to 9 years old, and 55+ years old, and the number of cases decreased with age (Fig. 5G and 5H).

**Figure 6.**
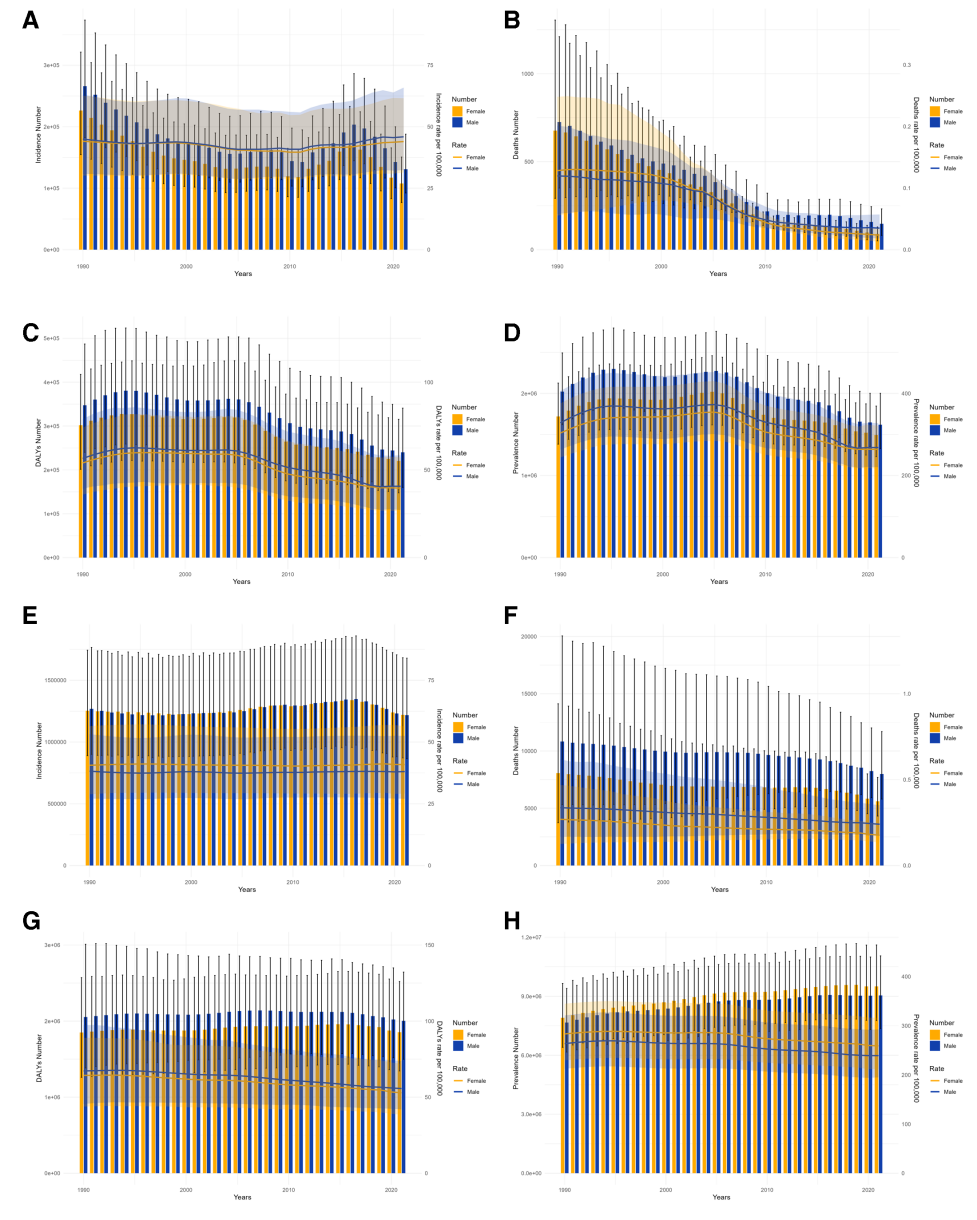
Comparison of full-age cases and age-standardized rates of incidence, prevalence, mortality, and DALYs among men and women in China and globally from 1990 to 2019. (A) Incident cases and ASIR (China); (B) prevalent cases and ASPR (China); (C) death cases and ASMR (China); (D) DALYs counts and ASDR (China); (E) incident cases and ASIR (Global); (F) Prevalent cases and ASPR (Global); (G) Death cases and ASMR (Global); (H) DALYs counts and ASDR (Global). Bar charts represent counts (with 95% CI); lines represent age-standardized rates. ASDR = age-standardized DALY rate, ASPR = age-standardized prevalence rate, ASIR = age-standardized incidence rate, ASMR = age-standardized mortality rate, CI = confidence interval, DALYs = disability-adjusted life years.

Figure 6 presents the comparison of disease burden and ASRs of CMLA in males and females of all age groups in China and globally from 1990 to 2021. Figure 6A shows the ASIR of CMLA for males and females, which peaked in 1990 and then decreased as the years increased, peaking again in 2016 and then decreasing again year by year. The incidence was consistently higher in men than in women, and the magnitude of the difference was consistently similar between men and women. ASPR of CMLA increased gradually from 1990 to 2005 in both men and women and decreased gradually after 2005. ASPR was consistently higher in men than in women (Fig. 6D). Unlike the trend in China, the ASPR of CMLA for both males and females worldwide increased over the years, while the ASIR level remained basically stable amid fluctuations (Fig. 6E, 6H). Furthermore, Figure 6C shows that the ASDR of CMLA differed between men and women at all ages in 1990, with men consistently having higher ASDR than women. Over time, the difference between the sexes gradually narrowed, and the overall ASDR showed a downward trend. The ASMR of CMLA decreased significantly with the increase in years and leveled off after 2010 (Fig. 6B). Globally, ASDR and ASMR levels were generally stable across age groups (Fig. 6G, 6F).

### 3.6. Auto-regressive integrated moving average model

**Figure 7.**
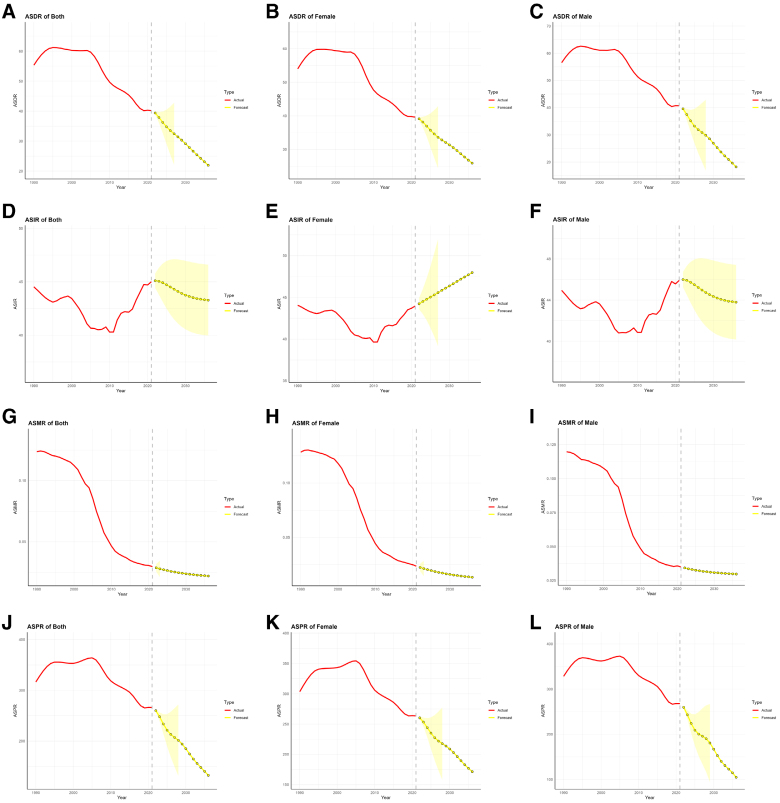
Predicted trends of SCI ASIR and ASPR in China over 15 years (2022–2036). The red lines represent the true trend of age-standardized incidence and prevalence of CMLA during 1990–2021; the yellow lines represent the predicted trend, and the light-yellow shaded regions represent the 95% confidence interval of predicted values; the gray dotted vertical line splits data into true value (1990–2021) and predicted value. (A) ASDR of both; (B) ASDR of female; (C) ASDR of male; (D) ASIR of both; (E) ASIR of female; (F) ASIR of male; (G) ASMR of both; (H) ASMR of female; (I) ASMR of male; (J) ASPR of both; (K) ASPR of female; (L) ASPR of male. ASDR = age-standardized DALY rate, ASPR = age-standardized prevalence rate, ASIR = age-standardized incidence rate, ASMR = age-standardized mortality rate, CMLA = congenital musculoskeletal and limb anomalies, DALYs = disability-adjusted life years.

KPSS stationarity test showed that part of the original sequence was non-stationary (*P* < .05), and the sequence was stationary after difference (*P* > .05; Table S2, Supplemental Digital Content 2). Table 3 shows the optimal ARIMA model parameters and corresponding Akaike information criterion and Bayesian information criterion values automatically selected according to corrected Akaike information criteria. The Ljung‑Box test of the residual showed that the residual of each model was white noise (*P* > .05), and ACF and Q‑Q plot further confirmed that the model was reasonable (Figure S1, Supplemental Digital Content 3). Overall, ASIR, ASPR, ASDR, and ASMR are all expected to decline in China over the next 15 years (Fig. 7A, 7D, 7G, 7J).

**Table 3:** ARIMA model parameters and residual diagnostics for ASRs.

Gender	ASRs	(p, d, q)	AIC	BIC	AICc	Ljung-Box χ^2^ (lag = 10)	*P* value	White noise (*P* > .05)
Both	ASIR	(2, 0, 0)	30.93	36.8	32.41	7.83	.65	Yes
	ASPR	(2, 2, 1)	104.11	109.72	105.71	5.33	.87	Yes
	ASMR	(1, 1, 0)	−307.03	−304.16	−306.6	8.63	.57	Yes
	ASDR	(2, 2, 0)	15.59	19.8	16.52	3.73	.96	Yes
Male	ASIR	(2, 0, 0)	37.88	43.74	39.36	8.28	.6	Yes
	ASPR	(2, 2, 1)	111.22	116.82	112.82	6.15	.8	Yes
	ASMR	(1, 1, 0)	−304.76	−301.9	−304.34	10.68	.38	Yes
	ASDR	(2, 2, 0)	22.49	26.69	23.41	4.13	.94	Yes
Female	ASIR	(0, 2, 0)	22.77	24.17	22.91	13.27	.21	Yes
	ASPR	(2, 2, 0)	97.58	101.79	98.51	7.24	.7	Yes
	ASMR	(1, 1, 0)	−303.38	−300.52	−302.96	8.37	.59	Yes
	ASDR	(3, 2, 0)	12.49	18.09	14.09	3.08	.98	Yes

AIC = Akaike information criterion, AICc = corrected Akaike information criterion, ARIMA = auto-regressive integrated moving average, ASDR = age-standardized DALY rate, ASPR = age-standardized prevalence rate, ASIR = age-standardized incidence rate, ASMR = age-standardized mortality rate, ASR = age-standardized rate, BIC = Bayesian information criterion.

When making gender-stratified predictions of ASR in China, it is expected that the ASDR and ASPR of males and females will decline in the next 15 years (Fig. 7B, 7C, 7K, 7L), and the decline in ASDR and ASPR of males will be more significant (Fig. 7C, 7L). For ASIR predictions, it is expected that the ASIR of males will first decline and then stabilize, while the ASIR of females is projected to gradually increase over the next 15 years (Fig. 7E). In the next 15 years, the ASMR of males and females will decline slowly (Fig. 7H, 7I).

### 3.7. Decomposition analysis

**Figure 8.**
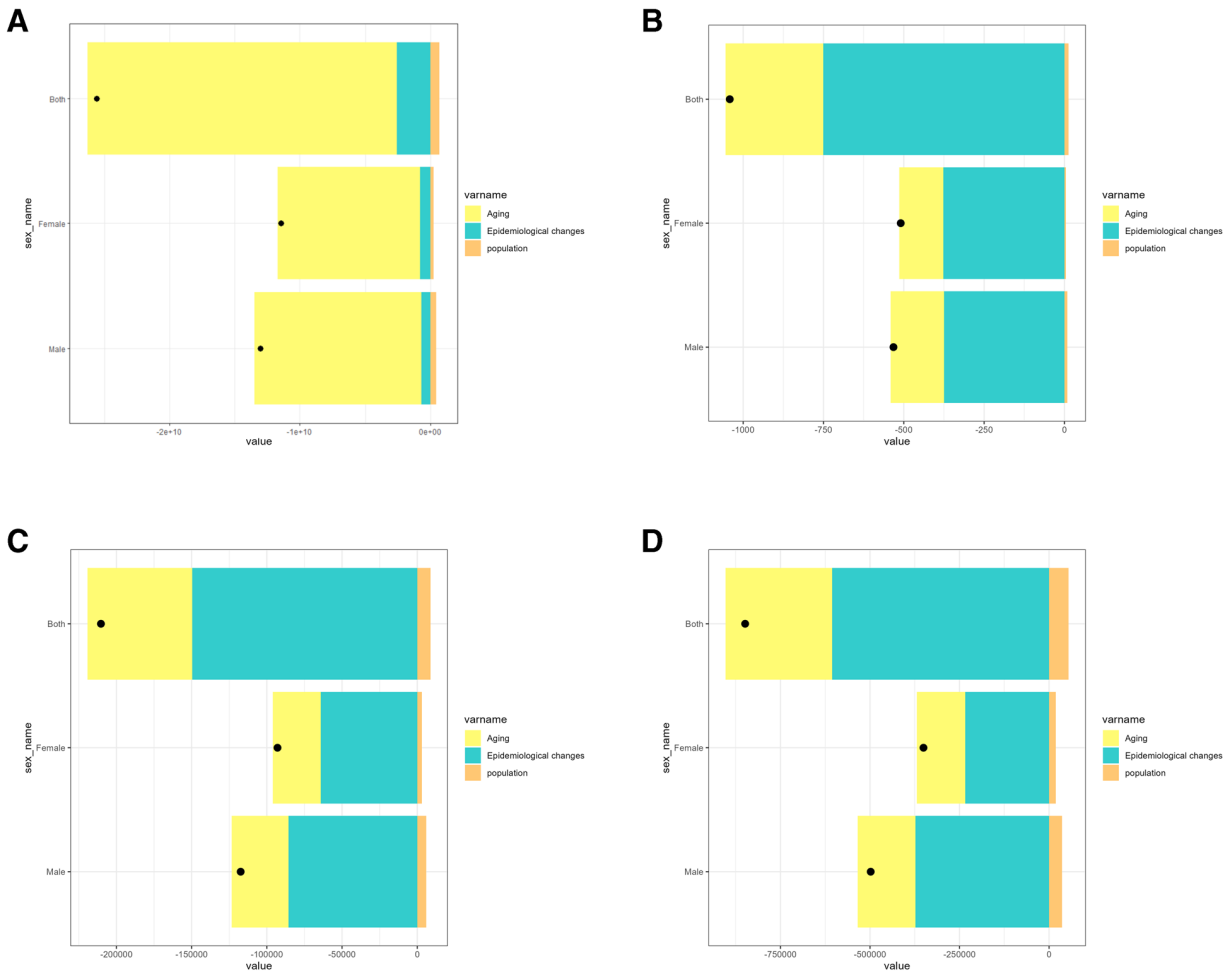
ASR changes in CMLA according to population growth, aging, and population-level determinants. ASR changes of global and Chinese CMLA from 1990 to 2021, black dots represent the overall value of the variables contributed by all 3 components. For each component, the magnitude of a positive value indicates that the component drives a corresponding increase in ASR. The magnitude of the negative value indicates that the component causes a corresponding reduction in ASR. (A) ASIR, (B) ASMR, (C) ASDR, (D) ASPR. ASDR = age-standardized DALY rate, ASMR = age-standardized mortality rate, ASPR = age-standardized prevalence rate, ASR = age-standardized rates, ASIR = age-standardized incidence rate, CMLA = congenital musculoskeletal and limb anomalies, DALYs = disability-adjusted life years.

Decomposition analysis showed that over the past 30 years, the 4 indicators of ASR in China have declined significantly across the board, and the effects of aging, population, and epidemiological changes on different indicators and gender stratification are different (Fig. 8). Epidemiological changes were the most important factors contributing to the decline of all ASRs. Intuitively, the aging effect is the primary factor affecting the incidence of CMLA. Population increase was always detrimental to the decline of ASR, and this effect was particularly evident in ASIR. Aging and epidemiological changes always play a favorable and decisive role in the decline of ASR (Table 4).

**Table 4:** Results of decomposition analysis.

	Sex	Overall_difference	Change due to population-level determinants (95% UI) [% contribute to the total changes (95% UI)]		
			Aging	Population	Epidemiological_changes
Male	−118,336.6	−37,406.75 (−51,301.91, −26,145.36) [31.58 (21.53, 49.96)]	5977.54 (−1573.00, 13,607.88) [−5.05 (−13.59, 1.28)]	−86,907.38 (−134,735.55, 43,949.29) [73.38 (56.59, 83.11)]
Female	−91,744.2	−31,830.38 (−43,644.88, −21,360.02) [34.63 (23.24, 56.15)]	3048.33 (−3605.19, 10,134.67) [−3.22 (−13.43, 3.70)]	−62,962.13(−106,630.47, −26,096.23) [68.79 (46.22, 80.50)]
Both	−210,199.4	−69,453.89 (−91,963.32, −49,080.17) [33.11 (21.70, 53.48)]	8895.65 (−6457.45, 24,114.46) [−4.32 (−14.23, 2.79)]	−149,641.17 (−238,897.43, −68,761.28) [71.28 (53.88, 81.94)]
ASIR	Male	−130,065.38	−127,991.57 (−169,963.33, −94,178.54) [97.63 (56.40, 344.34)]	4698.21 (−1718.43, 11,306.25) [−3.48 (−15.84, 2.01)]	−6772.02 (−102,287.16, 91,240.26) [6.07 (−230.09, 45.83)]
	Female	−114,488.53	−109,242.33 (−145,449.64, −80,091.33) [95.02 (55.31, 286.10)]	2428.46 (−2702.08, 7832.52) [−2.09 (−10.49, 2.96)]	−7674.67 (−94,691.35, 73,023.69) [7.06 (−182.55, 46.03)]
	Both	−255,671.38	−237,214.77 (−321,542.43, −174,010.19) [91.72 (55.03, 321.74)]	7210.25 (−4900.24, 19,267.93) [−2.84 (−13.36, 2.17)]	−25,666.86 (−212,854.14, 171,606.56) [10.39 (−214.46, 46.17)]
ASMR	Male	−520.28	−166.91 (−271.88, −99.94) [30.77 (24.99, 44.36)]	8.93 (−2.27, 23.05) [−1.68 (−4.34, 0.47)]	−367.29 (−680.77, −170.76) [70.93 (58.59, 76.29)]
	Female	−488.19	−131.56 (−230.28, −76.49) [26.93 (22.65, 33.41)]	4.15 (−4.90, 14.66) [−0.85 (−2.98, 1.01)]	−360.79 (−683.99, −186.01) [73.96 (67.51, 78.08)]
	Both	−1013.19	−298.15 (−472.64, −194.92) [29.12 (24.52, 36.29)]	12.69 (−9.85, 36.98) [−1.29 (−3.55, 0.88)]	−727.73 (−1226.01, −421.78) [72.20 (65.08, 76.51)]
ASPR	Male	−502,886.08	−160,373.85 (−208,034.14, −119,528.17) [32.01 (21.91, 49.42)]	36,681.90 (−9773.00, 82,591.87) [−7.41 (−19.61, 1.68)]	−379,194.13 (−540,694.73, −217,469.15) [75.18 (60.20, 85.62)]
	Female	−349,323.31	−135,982.76 (−176,238.65, −96,735.55) [38.80 (25.67, 63.76)]	18,764.63 (−21,972.31, 61,166.90) [−5.27 (−22.11, 5.71)]	−232,105.17 (−381,469.93, −92,042.08) [66.61 (42.95, 80.53)]
	Both	−850,038.77	−297,157.79 (−385,916.55, −218,692.66) [35.16 (23.23, 57.31)]	54,799.04 (−40,091.49, 146,389.45) [−6.62 (−21.78, 4.16)]	−607,680.02 (−939,827.53, −291,470.14) [71.75 (53.80, 83.76)]

ASDR = age-standardized DALY rate, ASPR = age-standardized prevalence rate, ASIR = age-standardized incidence rate, ASMR = age-standardized mortality rate, UI = uncertainty intervals.

## 4. Discussion

Based on the data from the GBD 2021 database, this study comprehensively evaluated the incidence, prevalence, mortality, and DALYs of CMLA in China and worldwide. The Joinpoint and APC models were used to analyze the trends. The ARIMA model was used to predict the disease burden of CMLA in the next 15 years. Finally, the contribution of different factors to the disease burden was evaluated by decomposition analysis. The results showed that the prevalence, incidence, mortality, and DALY of CMLA in China decreased significantly from 1990 to 2021. During the past 30 years, the ASRs of each index remained declining or relatively stable.

The burden of disease in CMLA has declined over the past 3 decades as global living standards have improved and healthcare has become more accessible. The results of this study showed that between 1990 and 2021, the incidence in China decreased by 51.54%, while it was only 3.32% globally. The promotion of prenatal ultrasound in China occurred during the same period as the acceleration of the decline in domestic incidence after 2000. From 2010 to 2019, ASIR increased, accompanied by the progress of diagnostic technology, the intensification of screening, the improvement of prenatal ultrasound sensitivity, and the expansion of the newborn screening monitoring network. On the contrary, it may be the increased proportion of advanced maternal age pregnancies and the prevalence of maternal metabolic diseases.^[11,12]^

However, the global ASIR has stagnated or even increased slightly in fluctuations, which may be due to the dynamic counterbalance of improved diagnostic capacity and increased environmental risks across regions. Low- and middle-income countries continue to release historically precipitable cases through prenatal ultrasound and genetic testing, while high-income countries have reached a plateau.^[3]^ At the same time, irreversible exposure to pollutants and the surge of maternal metabolic diseases pose upward pressure on the true incidence.^[13–15]^ In terms of offsetting mechanisms, prenatal diagnosis and termination of pregnancy are effective in high-income regions to hedge environmental risks,^[16,17]^ More than 80% of the world’s population is located in low- and middle-income countries, resulting in a macro-statistical balance after population weighting. It should be warned that when the diagnostic dividend gradually saturates, such as the European ASIR entering the volatility range in recent years,^[18,19]^ or environmental exposure such as PM2.5 breaching the biological compensation threshold,^[20,21]^ this equilibrium state has significant vulnerabilities that may trigger the global ASIR into the upward channel. It is worth noting that if an increase in ASIR is observed without a synchronous change in perinatal mortality, it is necessary to be alert to a false increase dominated by increased diagnostic sensitivity. A concomitant increase in the proportion of major malformations suggests an increased true risk exposure. It is suggested to analyze the prenatal diagnosis rate, pregnancy termination rate, and malformation spectrum of live births in combination to debias the estimation.

The overall prevalence of CMLA in China decreased by 16.79% in the past decade, but there was significant age-stratified heterogeneity. The proportion of children below 29 years old decreased significantly, and the early intervention system in China continued to improve during the same period. In 2021, the coverage of children’s health services in China had reached more than 85%. In contrast, the 37% increase in the proportion of patients 30 years of age or older was probably driven by 2 factors. First, the survivor effect has significantly prolonged the survival period of patients due to the improvement of medical care. Second, the accumulation of uncorrected secondary injuries to CMLA during childhood can lead to aggravated conditions in adulthood or cause other secondary diseases. For instance, children with congenital foot or hip joint abnormalities have a significantly increased risk of developing secondary osteoarthritis in adulthood.^[22,23]^ At the global level, unlike in China, the overall prevalence increased by 19.13%, which was mainly attributed to the backlog of cases caused by the shortage of medical resources in low- and middle-income countries.^[24]^ This divergence highlights the advantages of high-income countries in life cycle health management and warns that low- and middle-income regions need to strengthen the construction of deformity rehabilitation systems.

The CMLA crude death rate in China showed an accelerating improvement trend, with a decline of 83.02% from 1990 to 2021, while the global decline was only 28.05% during the same period. Since orthopedic surgery was included in China’s national insurance program in 2009, there has been a surge in surgical rates in rural areas. Combined with thousands of operations supported by the National Public Welfare Fund, this direct intervention has helped accelerate the reduction in infant mortality by reducing malformation-related complications and perioperative deaths. The effect of the intervention was further supported by the changes in the age distribution of death in China. The proportion of deaths in children below 1 year old had declined significantly, which marked a historic breakthrough in the treatment of severe neonatal diseases, and the death spectrum was shifting from infancy to adulthood. However, we noted that the decline of ASMR in China was slower, which may be due to the transition to an aging-dominated society, and the decline was offset by the expansion of the elderly population.

The DALYs in the CMLA in China showed significant sex–age interaction, with an overall decline of 29.04% (only 1.17% globally). Among children below 4 years old, early surgical intervention coincided with the maximum reduction in DALYs, and the perinatal health care system also made progress during the same period. The gender difference in DALYs peaked among adolescents aged 15 to 19 years (male/female = 1.24 in 1990, male/female = 1.23 in 2021), reflecting the lack of sex-specific prevention. Insufficient access to rehabilitation services and the accumulation of secondary injuries in people aged 55 and over contributed to the second peak of DALYs. Deep attribution analysis shows that the innovation of prenatal diagnosis technology and the popularization of neonatal intensive care have significantly reduced the disability severity of congenital malformations, while the prevention and control of infectious diseases have reduced the complications related to malformations from the source.^[25–27]^ This life-cycle intervention model may be the key to the fact that China’s DALYs decline is much higher than the global average.

It should be emphasized that all the above observations are based on population ecological-level data and cannot be directly extrapolated to the individual level, so ecological fallacies should be guarded against. In addition, all the above observations are on time trends, and it is not possible to establish a causal relationship between specific policy changes and the improvement of burden indicators.

It is worth noting that when observing ASIR, ASPR, ASMR, and ASDR indicators in China and the world, we found that these indicators seem to have increased to varying degrees since 2019. Due to the short observation period (2019–2021), it is not yet possible to judge whether this is caused by the epidemic of COVID-19. While the COVID-19 pandemic is temporally consistent with this trend, it differs from the trend shown during the global SARS epidemic in 2002–2003. According to the above burden and time trends, the epidemic period of SARS did not cause significant changes in the disease burden of CMLA in China and the world. Therefore, based on the available data, it is not possible to conclude that COVID-19 infection itself or its indirect effects, such as the run on health care resources, disruptions to routine health care services, and changes in social behavior, are directly attributable to the observed rise in disease burden. However, an association between viral infection during pregnancy and congenital malformations has been reported.^[28–30]^ The possible association of COVID-19 with congenital abnormalities of the upper limbs has also given researchers pause for thought.^[31]^ This may make it urgent for us to systematically evaluate whether COVID-19 infection may become a novel and important risk factor for CMLA in the post-pandemic era.

We have noticed that the burden on males is generally higher than that on females. This observation may be influenced by the confounding effect of a larger male population in certain age groups. After standardized correction by age and gender stratification, in almost all age groups, the burden of males is still higher than that of females. These findings seem to suggest that the observed male burden may be due to genuine differences in biological susceptibility between genders. First, due to the variations in XY chromosomes between males and females, males are more likely to exhibit diseases or CMLA due to gene mutations or defects on the X chromosome.^[32,33]^ Second, some studies have reported that male fetuses may be more sensitive to adverse environmental stresses such as malnutrition, alcohol abuse, and infection, or to the mother’s health condition in the womb.^[34]^ The ARIMA model showed that the incidence, prevalence, mortality, and DALYs of CMLA in China would generally decrease from 2022 to 2036. ASPR, ASDR, and ASMR form a coherent trajectory with the historical downward trend and are expected to continue to decline in the future. Male ASIR may decline after the upward period from 2011 to 2021, while female ASIR may continue to rise. All these suggest that the burden of CMLA between men and women has been and will be uneven. In the post-pandemic context described above, enhanced prospective research on potential long-term health consequences in specific vulnerable populations, such as pregnant women, may help better address the burden of CMLA disease.

The results of this study may provide valuable data to health authorities and may contribute to the reduction of the CMLA burden globally. However, this study has several limitations that need to be acknowledged. First, GBD estimates depend on the quality and quantity of data, such as disease diagnosis and the measurement of environmental risk factors over time. In areas with limited healthcare access and economically disadvantaged populations, incomplete disease diagnosis and fragmented vital registration systems may contribute to inadequate CMLA screening in these areas. Thus, CMLA burden data may be underestimated. In addition, although the definition of CMLA used in this study excludes definitive chromosomal syndromes, some mild or undiagnosed syndromes may be misclassified as isolated CMLA. There may be residual confounding due to incomplete coding or incomplete clinical documentation, or potential bias due to misclassification and miscoding of disorders. Second, diagnostic and testing methods for CMLA may have changed over time, and differences in data collection and diagnostic tools in different periods may also lead to potential bias in the data. Therefore, these national data cannot be directly used as the specific reference level of disease burden in specific regions. Further in-depth analyses based on data specific to each region are also needed. The ARIMA model’s reliance on historical trends limits its sensitivity to emergencies, such as healthcare fragmentation during the COVID-19 pandemic, which may affect forecast accuracy beyond 2021. In addition, ARIMA’s assumption of linear temporal patterns may not capture the complex interactions between demographic changes and epidemiological transitions, especially in rapidly developing regions.^[35]^ Because the Joinpoint regression analysis was performed using single-point estimates of ASRs, which do not support uncertainty weighting per se, the UIs provided by the GBD were not formally incorporated. While this practice is common in GBD-based trend studies, it can lead to underestimation of the true variability of trend estimates.

This study presents CMLA data with a holistic perspective of China to describe the overall burden of CMLA on a China-wide scale. This study did not obtain data on the disease burden in different regions of China, and the level of disease burden in different regions may vary due to specific factors such as the level of socioeconomic development, geographical environmental factors, genetics, ethnicity, medical resources, and vaccine coverage.^[36]^ Specifically, in terms of urban–rural differences, prenatal ultrasound coverage and diagnostic accuracy in urban areas are much higher than those in rural areas, which may lead to underreporting and delayed diagnosis of CMLA in rural areas, thus underestimating the true disease burden. In terms of regional differences, access to perinatal care, surgery, and rehabilitation resources in the western underdeveloped provinces was significantly lower than that in the eastern coastal areas, so the national average data may mask the reality of high burden and low diagnosis in the western region. Based on the above limitations, it is recommended that future studies make full use of the provincial birth defects surveillance network to analyze the disease burden at the sub-national level in order to more precisely reveal the differences in disease burden among different regions in China.

Finally, our investigation was an examination of the database data of GBD 2021. Therefore, although the results of this study could not be externally checked, the quality and quantity of the accessible data greatly supported the accuracy of the results.

### 4.1. Implications for public health

The results of this study have far-reaching implications for the development of epidemiology worldwide and in China. First, the “age migration” of disease burden revealed by the study, that is, the increase in the cumulative burden of adult patients, urgently calls for epidemiological research to shift from the traditional, child-centered disease distribution model to the continuous monitoring and management of the disease process covering the whole life cycle. This means that epidemiological strategies need to strengthen the study of the distribution and influencing factors of occupational health risks, psychosocial factors, and secondary bone and joint problems in adult patients, so as to reveal the evolution trajectory of the disease burden more comprehensively. Second, China’s success in reducing the burden of disease highlights the value of bringing forward the threshold of epidemiological interventions and integrating them deeply into the public health system. The effectiveness of early screening and early intervention in young children has confirmed that population-based epidemiological surveillance and early intervention are the core links to reduce the long-term disability rate and improve the quality of life of patients with CMLA. Therefore, improving the accessibility of rehabilitation services and building an epidemiological surveillance and intervention network from community to professional institutions are the keys to reducing the overall burden of disease worldwide, especially in low- and middle-income areas. In addition, the environmental and lifestyle factors pointed out in the study also suggest that epidemiological research should not only be limited to the distribution and etiology of diseases but also be extended to population research on the health behaviors of patients and their families, the application effect of assistive technology, and the need for environmental modification, so as to achieve comprehensive health risk assessment, prevention, and control from the biopsychosocial medical model. In the future, advanced technologies such as genetic technology, regenerative medicine, and intelligent assistive devices should be actively introduced into the field of epidemiology, and successful public health experience should be translated into generalizable epidemiological practice, so as to eventually build a powerful and universal lifelong health monitoring and support system, providing solid epidemiological evidence and strategies to support the vision of a healthy start in life for every child.

## 5. Conclusion

This study comprehensively evaluated the dynamic changes and future trends of the disease burden of CMLA in China and worldwide from 1990 to 2021. The study found that the burden of CMLA has been significantly reduced in China, while global progress has been relatively slow and uneven. The structure of the disease burden showed obvious age migration characteristics and gender differences, and its changing trend was driven by multiple factors. This study provides an important reference for health policymakers on the current status of the CMLA burden and its drivers. The results of this study not only show the value of successful experiences in China, such as strengthening prenatal screening, improving early childhood intervention, and lifelong rehabilitation systems, but also warn the world to pay more attention to the insufficient access to medical resources and the increasing cumulative burden of adult patients in low- and middle-income regions. Short-term fluctuations and new challenges remind us that efforts to reduce CMLA need to be sustained. In the future, we should continue to strengthen maternal and neonatal healthcare measures, promote the application of more advanced medical technology and diagnostic methods, and pay attention to the optimization of the environment and lifestyle, so as to provide every child with a healthy start in life. At the same time, this study provides key enlightenment for the rehabilitation strategy of CMLA. Rehabilitation services need to be transformed to the “whole life cycle” model, and the intervention threshold should be moved forward. China’s successful experience has proved that deep integration of rehabilitation into the public health system is an effective way to reduce the long-term burden. In the future, efforts should be made to improve the accessibility and continuity of rehabilitation services to meet the ongoing challenges worldwide.

## Acknowledgments

We thank the Institute for Health Metrics and Evaluation (IHME) for providing open access.

## Author contributions

**Methodology:** Min-cheng Zou.

**Data curation:** Wen-dong Liu.

**Conceptualization:** Xiao-dong Wang, Guang-hao Su.

**Funding acquisition:** Ya Liu.

**Writing – review & editing:** Guang-hao Su, Ya Liu.

**Writing – original draft:** Min-cheng Zou, Wen-dong Liu.





**Figure:**