# Supplementary material for: Analysis and comparison of the trends in burden of congenital musculoskeletal and limb anomalies in China and worldwide from 1990 to 2021
Source: Medicine (Baltimore). 2026 Jun 12;105(24):e49244. doi: 10.1097/MD.0000000000049244 (PMC13268457; doi:10.1097/MD.0000000000049244)
Supplement: Supplementary file 1 [file medi-105-e49244-s001.docx]

| Age group (years) | Year | Proportion of population (M/F) | Prevalence (M/F) | Prevalence_correction (M/F) | DALYs (M/F) | DALYs_correction (M/F) | Mortality (M/F) | Mortality_correction (M/F) | Incidence (M/F) | Incidence_correction (M/F) |
| --- | --- | --- | --- | --- | --- | --- | --- | --- | --- | --- |
| <1 | 1990 | 1.15 | 1.17 | 1.02 | 1.13 | 0.98 | 1.10 | 0.97 | 1.18 | 1.02 |
|  | 2021 | 1.16 | 1.21 | 1.04 | 1.34 | 1.53 | 1.68 | 1.44 | 1.22 | 1.05 |
| 2-4 | 1990 | 1.12 | 1.26 | 1.12 | 1.13 | 1.01 |  |  |  |  |
|  | 2021 | 1.15 | 1.21 | 1.05 | 1.14 | 0.99 |  |  |  |  |
| 5-9 | 1990 | 1.08 | 1.26 | 1.17 | 1.26 | 1.16 |  |  |  |  |
|  | 2021 | 1.14 | 1.20 | 1.05 | 1.18 | 1.03 |  |  |  |  |
| 10-14 | 1990 | 1.07 | 1.22 | 1.14 | 1.23 | 1.15 |  |  |  |  |
|  | 2021 | 1.14 | 1.20 | 1.06 | 1.20 | 1.05 |  |  |  |  |
| 15-19 | 1990 | 1.06 | 1.19 | 1.12 | 1.24 | 1.17 |  |  |  |  |
|  | 2021 | 1.16 | 1.20 | 1.04 | 1.23 | 1.06 |  |  |  |  |
| 20-24 | 1990 | 1.05 | 1.12 | 1.07 | 1.14 | 1.09 |  |  |  |  |
|  | 2021 | 1.13 | 1.13 | 1.00 | 1.14 | 1.01 |  |  |  |  |
| 25-29 | 1990 | 1.06 | 1.12 | 1.06 | 1.11 | 1.05 |  |  |  |  |
|  | 2021 | 1.12 | 1.10 | 0.98 | 1.08 | 0.97 |  |  |  |  |
| 30-34 | 1990 | 1.09 | 1.15 | 1.06 | 1.16 | 1.06 |  |  |  |  |
|  | 2021 | 1.07 | 1.05 | 0.98 | 1.06 | 0.99 |  |  |  |  |
| 35-39 | 1990 | 1.07 | 1.13 | 1.06 | 1.14 | 1.07 |  |  |  |  |
|  | 2021 | 1.05 | 1.03 | 0.98 | 1.03 | 0.98 |  |  |  |  |
| 40-44 | 1990 | 1.10 | 1.17 | 1.06 | 1.18 | 1.07 |  |  |  |  |
|  | 2021 | 1.05 | 1.03 | 0.98 | 1.03 | 0.98 |  |  |  |  |
| 45-49 | 1990 | 1.12 | 1.18 | 1.05 | 1.18 | 1.06 |  |  |  |  |
|  | 2021 | 1.03 | 1.00 | 0.98 | 1.00 | 0.98 |  |  |  |  |
| 50-54 | 1990 | 1.12 | 1.19 | 1.06 | 1.19 | 1.06 |  |  |  |  |
|  | 2021 | 1.02 | 1.00 | 0.98 | 1.00 | 0.98 |  |  |  |  |
