# Supplementary material for: Analysis and comparison of the trends in burden of congenital musculoskeletal and limb anomalies in China and worldwide from 1990 to 2021
Source: Medicine (Baltimore). 2026 Jun 12;105(24):e49244. doi: 10.1097/MD.0000000000049244 (PMC13268457; doi:10.1097/MD.0000000000049244)
Supplement: Supplementary file 2 [file medi-105-e49244-s002.docx]

| Indicator | | Series tested | KPSS statistic | p-value | Stationary (p>0.05) |
| --- | --- | --- | --- | --- | --- |
| ASIR | Both | Original (d=0) | 0.23 | >0.1 | Yes |
|  | Male | Original (d=0) | 0.21 | >0.1 | Yes |
|  | Female | 2nd differenced (d=2) | 0.05 | >0.1 | Yes |
| ASPR | Both | 2nd differenced (d=2) | 0.21 | >0.1 | Yes |
|  | Male | 2nd differenced (d=2) | 0.21 | >0.1 | Yes |
|  | Female | 2nd differenced (d=2) | 0.2 | >0.1 | Yes |
| ASDR | Both | 2nd differenced (d=2) | 0.26 | >0.1 | Yes |
|  | Male | 2nd differenced (d=2) | 0.25 | >0.1 | Yes |
|  | Female | 2nd differenced (d=2) | 0.27 | >0.1 | Yes |
| ASMR | Both | 1st differenced (d=1) | 0.24 | >0.1 | Yes |
|  | Male | 1st differenced (d=1) | 0.21 | >0.1 | Yes |
|  | Female | 1st differenced (d=1) | 0.26 | >0.1 | Yes |
